# WM130 preferentially inhibits hepatic cancer stem-like cells by suppressing AKT/GSK3β/β-catenin signaling pathway

**DOI:** 10.18632/oncotarget.12822

**Published:** 2016-10-22

**Authors:** Chen-Xu Ni, Yang Qi, Jin Zhang, Ying Liu, Wei-Heng Xu, Jing Xu, Hong-Gang Hu, Qiu-Ye Wu, Yan Wang, Jun-Ping Zhang

**Affiliations:** ^1^ School of Pharmacy, Second Military Medical University, Shanghai 200433, China; ^2^ Department of Pharmacy, Shanghai East Hospital, Tongji University, Shanghai 310000, China

**Keywords:** matrine derivative, hepatocellular carcinoma, cancer stem-like cells, GSK3β, AKT

## Abstract

The eradication of cancer stem cells (CSCs) is significant for cancer therapy and prevention. In this study, we evaluated WM130, a novel derivative of matrine, for its effect on CSCs using human hepatocellular carcinoma (HCC) cell lines, their sphere cells, and sorted EpCAM^+^ cells. We revealed that WM130 could not only inhibit proliferation and colony formation of HCC cells, but also suppress the expression of some stemness-related genes and up-regulate some mature hepatocyte marker genes, indicating a promotion of differentiation from CSCs to hepatocytes. WM130 also suppressed the proliferation of doxorubicin-resistant hepatoma cells, and markedly reduced the cells with CSC biomarker EpCAM. Moreover, WM130 suppressed HCC spheres, not only primary spheres but also subsequent spheres, indicating an inhibitory effect on self-renewal capability of CSCs. Interestingly, WM130 exhibited a remarkable inhibitory preference on HCC spheres and EpCAM^+^ cells rather than their parental HCC cells and EpCAM^−^ cells respectively. *In vivo*, WM130 inhibited HCC xenograft growth, decreased the number of sphere-forming cells, and remarkably decreased the levels of *EpCAM* mRNA and protein in tumor xenografts. Better inhibitory effect was achieved by WM130 in combination with doxorubicin. Further mechanism study revealed that WM130 inhibited AKT/GSK3β/β-catenin signaling pathway. Collectively, our results suggest that WM130 remarkably inhibits hepatic CSCs, and this effect may via the down-regulation of the AKT/GSK3β/β-catenin pathway. These findings provide a strong rationale for the use of WM130 as a novel drug candidate in HCC therapy.

## INTRODUCTION

Liver cancer, including hepatocellular carcinoma (HCC), remains a leading cause of cancer death worldwide [1]. Increasing evidence indicates that a small subpopulation of cancer cells, termed cancer stem cells (CSCs), exist in many types of cancer [2–8]. Recently, the existence of CSCs has also been shown in liver cancer cell lines and primary HCC specimens [9–19]. HCC CSCs can be isolated and characterized by using various stem cell markers such as EpCAM, CD133, CD90 and CD44. They exhibit stem cell-like characteristics such as spherical colony formation *in vitro*, self-renewal, differentiation and resistance to chemo- and radiotherapies. CSCs are responsible for cancer initiation, growth, metastasis and recurrence, and have been regarded as a critical target for cancer eradication [20]. Actually, neutralizing antibodies, RNA interference against CSC markers, and small molecule CSC inhibitors alone or in combination with chemotherapies have been shown to reduce tumor incidence, growth, and metastasis in animal models [10, 14, 19–24]. Thereby, therapy specifically eradicating CSCs, either alone or in combination with conventional chemotherapies, may provide advantages for cancer eradication.

**Figure 1:**
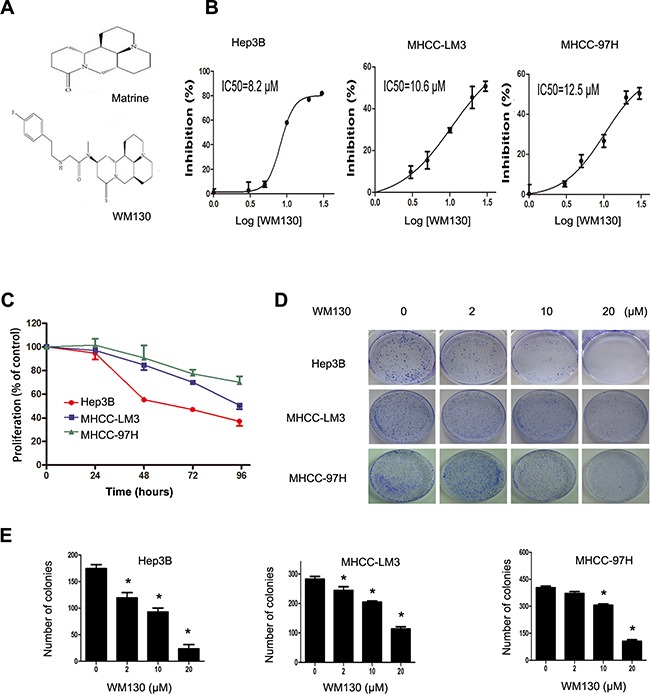
Effect of WM130 on the proliferation and colony formation of human hepatoma cells **A.** Chemical structures of matrine and its derivative WM130. **B.** WM130 inhibited the proliferation of human hepatoma cells. Hep3B, MHCC-LM3 and MHCC-97H cells were treated with WM130 for 72 h. Proliferation was measured by a CCK8 assay. The IC_50_ values are shown. **C.** WM130 inhibited the proliferation of human hepatoma cells in a time-dependent manner. **D** and **E.** WM130 suppressed the colony formation of human hepatoma cells. N=3, *p<0.05 versus control.

Matrine (Figure 1A), a major active alkaloid of the Chinese herbal medicine *Sophora flavescens Ait*, possesses significant anti-neoplastic, anti-inflammatory, anti-fibrotic, and anti-viral properties [25–31]. It has been used in clinic in China for the treatment of cancer, viral hepatitis, enteritis, viral myocarditis, arrhythmia, colpitis and eczema [32]. Nevertheless, its potency is relatively low and its half-life is relatively short. To improve its features as a drug, we have recently semi-synthesized a series of matrine derivatives by replacing the carbonyl oxygen atom with a sulfur atom and introducing various amino groups to the keto-beta position. These derivatives exhibit better anti-inflammatory, anti-fibrotic and anti-tumor activities [33–36]. WM130 (C_30_N_4_H_40_SO_5_F; Figure 1A) is one of the novel matrine derivatives with improved pharmacological activities. In this study, we examined WM130 for its effect on inhibiting hepatic cancer stem-like cells in both HCC cell lines and xenografts, and elucidated the underlying mechanism.

## RESULTS

### WM130 inhibits proliferation and colony formation of hepatoma cells

WM130 suppressed the proliferation of HCC cell lines, including Hep3B, MHCC-LM3 and MHCC-97H, in a concentration-dependent manner with IC_50_ values being 8.2, 10.6 and 12.5 μmol/L respectively (Figure 1B). Moreover, WM130 exerted its effect in a time-dependent manner (Figure 1C), and its anti-proliferative effect was enhanced with the passing of time.

We further examined the effect of WM130 on colony formation of hepatoma cells through clonogenic assay. The results indicated that WM130 significantly reduced the number of formed colonies, and the results are consistent in all the three HCC cell lines tested (Figure 1D and 1E).

Notably, the immortalized human hepatocyte cell line L02 and rat primary hepatocytes were not affected by WM130 at the concentrations tested (data not shown), which indicated that WM130 exerted a more selective activity against HCC cells than normal cells.

### WM130 inhibits proliferation of doxorubicin (DOX)-resistant hepatoma cells and reduces the expression of stemness-related genes in hepatoma cells

**Figure 2:**
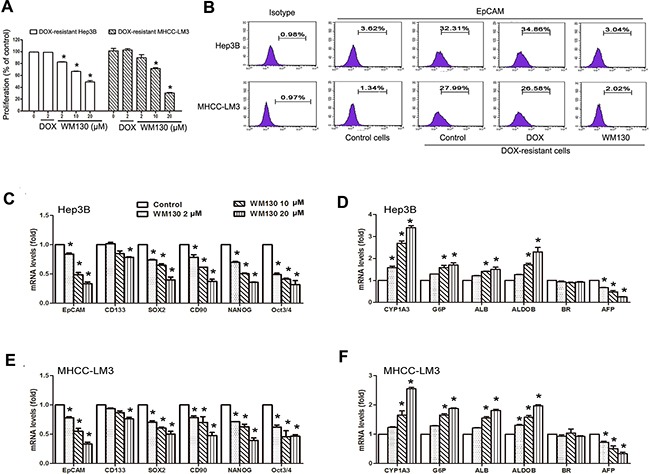
The effect of WM130 on DOX-resistant cells and the influence of WM130 on the expression of genes relevant to stemness and differentiation of hepatic stem/progenitor cells Hep3B and MHCC-LM3 cells were treated with DOX (2 μmol/L) for 5 days to obtain DOX-resistant cells, and the resistant cells were exposed to WM130 or DOX for 3 days. **A.** DOX-resistant HCC cells remained sensitive to WM130. N=3, *p<0.05 versus control. **B.** WM130 (10 μmol/L) eliminated the increased EpCAM^+^ fraction in DOX-resistant cells. The results are representative of two independent experiments. **C-F.** Hep3B (C, D) and MHCC-LM3 (E, F) cells were treated with 10 μmol/L WM130 for 24 h. The expression of “stemness” and liver-specific genes was detected by real-time RT-PCR. The mRNA levels were normalized against β-actin and are relative to the control. N=3, *p<0.05 versus control.

We further examined the effect of WM130 on DOX-resistant cells expressing CSC biomarker EpCAM. DOX-resistant HCC cells were obtained by exposing HCC cells (Hep3B and MHCC-LM3) to 2 μmol/L DOX for 5 days. The cells were resistant to DOX but still sensitive to WM130 (Figure 2A and Supplementary Figure S1A). Remarkably, among the DOX-resistant cells, the number of EpCAM^+^ cells was significantly increased and this increasing was eliminated by WM130 treatment (Figure 2B). More specifically, after WM130 treatments, the EpCAM^+^ cells were reduced from 32.32% to 3.04%, and from 27.99% to 2.02% in DOX-resistant Hep3B and MHCC-LM3 cells respectively. In contrast, no inhibitory effect was observed of DOX.

We further detected the expression of some stemness-related genes and mature hepatocyte biomarker genes in hepatoma cells. WM130 treatment concentration-dependently reduced the mRNA levels of stemness-related gene *EpCAM, CD133*, *CD90*, *Oct3/4, Sox2* and *NANOG* in HCC cells (Figure 2C and 2E, Supplementary Figure S1B). As to liver-specific genes, WM130 increased the expression of *cytochrome P450(CYP)1A3 (CYP1A3), glucose-6-phosphatase (G-6-P), albumin (ALB), aldolase B (ALDOB),* which was accompanied by the down-regulation of hepatocyte malignance gene *alpha-fetoprotein (AFP)*, while *biliverdin synthetase (BR)* expression remained unchanged (Figure 2D and 2F, Supplementary Figure S1C). These results suggest that WM130 may inhibit cancer stem-like cell and promote the differentiation from CSCs to hepatocytes.

### WM130 inhibits HCC spheres among hepatoma cells

**Figure 3:**
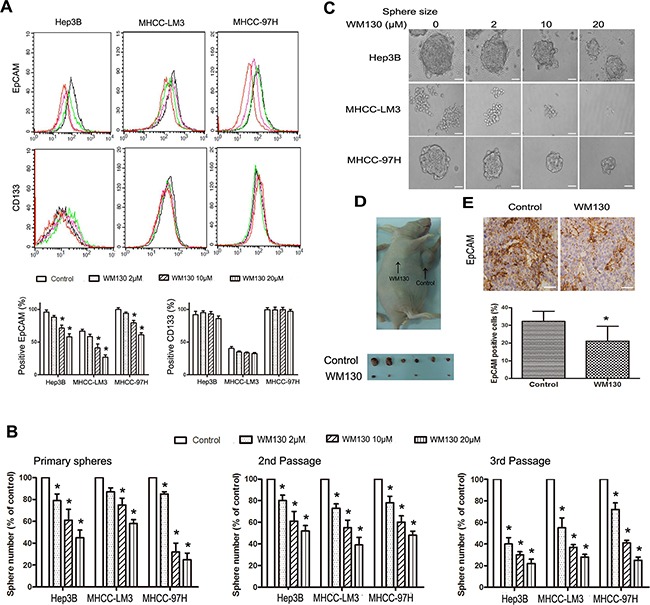
WM130 inhibits HCC spheres **A.** WM130 decreased the number of EpCAM^+^ cells in the spheres. The results are representative of three independent experiments (black line, control; green line, 2 μmol/L WM130; pink line, 10 μmol/L WM130; orange line, 20 μmol/L WM130). N=3, *p<0.05 versus control. **B.** WM130 reduced the number of Hep3B, MHCC-LM3 and MHCC-97H spheres. N=3, *p<0.05 versus control. **C.** WM130 reduced the size of primary spheres. Scale bar=50 μm. **D.** Images of the tumor xenografts derived from WM130-treated Hep3B spheres and control spheres. **E.** EpCAM expression in tumor tissues. Scale bar=100 μm. N=3, *p<0.05 versus control.

To determine the effect of WM130 on HCC spheres, we enriched populations of hepatic cancer stem-like cells using the sphere culture technique. Flow cytometric analysis revealed that WM130 treatment reduced the number of EpCAM^+^ cells in Hep3B, MHCC-LM3 and MHCC-97H spheres in a concentration-dependent manner, while WM130 rendered no evident influence on CD133^+^ cells (Figure 3A). WM130 concentration-dependently inhibited the formation of primary spheres, as evidenced by both reduced number and decreased size of the spheres (Figure 3B and 3C). Moreover, the number of subsequent spheres were also reduced under condition that WM130-treated primary spheres were cultured for subsequent two passages in the absence of WM130 (Figure 3B), indicating that WM130 inhibited the self-renewal ability of CSCs.

The inhibitory effect of WM130 on HCC spheres was confirmed in a xenograft model. WM130-treated Hep3B spheres and vehicle-treated spheres were subcutaneously injected into nude mice. Palpable tumors (about 1 mm in diameter) developed from vehicle-treated spheres within 2-3 weeks, while WM130 treatment delayed palpable tumor formation by about 2 weeks. The tumor xenografts derived from WM130-treated spheres were significantly smaller than those derived from control spheres (Figure 3D). Immunohistochemical staining revealed that the number of EpCAM^+^ cells was significantly diminished (*p* < 0.01) in the WM130 group compared with the control group (Figure 3E).

### WM130 preferentially inhibits HCC spheres and EpCAM^+^ Hep3B cells

**Figure 4:**
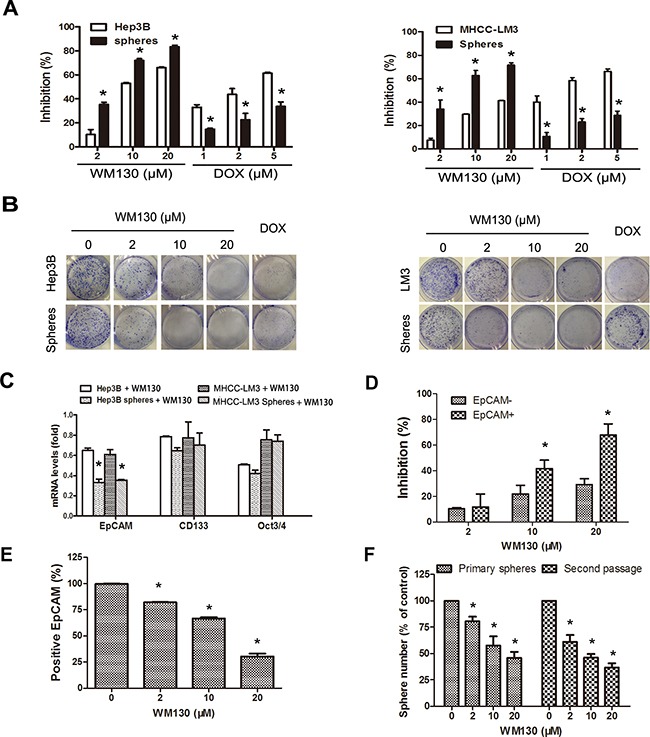
WM130 preferentially inhibits HCC spheres and the sorted EpCAM+ Hep3B cells **A.** Growth inhibitory effect of WM130 and DOX on Hep3B and MHCC-LM3 sphere cells compared with their corresponding parental cells. N=3, *p<0.05 versus control. **B.** Inhibition of colony formation by WM130 and DOX (2 μmol/L). N=3, *p<0.05 versus control. Quantitative data were shown in Figure S2B. **C.** WM130 preferentially decreased EpCAM expression in spheres. N=3, *p<0.05 versus control. **D.** WM130 preferentially inhibited proliferation of EpCAM^+^ Hep3B cells. N=3, *p<0.05 versus EpCAM^−^ Hep3B cells. **E.** WM130 reduced the number of EpCAM^+^ Hep3B cells. N=3, *p<0.05 versus control. **F.** WM130 reduced the number of EpCAM^+^ Hep3B spheres. Second passage of spheres was cultured without WM130. N=3, *p<0.05 versus control.

We further compared the inhibitory effect of WM130 on sphere cells and their corresponding parental HCC cells. WM130 preferentially inhibited sphere cell proliferation and colony formation in all the cell lines tested, including Hep3B, MHCC-LM3 and MHCC-97H. In contrast, DOX preferentially inhibited the HCC cells rather than their spheres (Figure 4A, 4B and Supplementary Figure S2A and S2B). Moreover, WM130 preferentially decreased the EpCAM mRNA in all the three types of spheres than in their parental cells. Nevertheless, as to the influence of WM130 on the expression of *CD133* and *Oct3/4*, no preference was observed on spheres or their parental cells (Figure 4C and Supplementary Figure S2C).

We fractionated Hep3B cells into EpCAM^+^ and EpCAM^−^ subpopulations by magnetic-activated cell sorting, with more than 99% purity in EpCAM^+^ cells and more than 82% purity in EpCAM^−^ cells (Supplementary Figure S3A). The sorted EpCAM^+^ Hep3B cells exhibited stronger proliferation ability and sphere forming ability than EpCAM^−^ cells (Supplementary Figure S3B and S3C). We generated single-cell derived clones by introducing EpCAM^+^ Hep3B cells into 96-well plates at a dilution yielding an average of <1 cell per well. The limiting dilution assay results indicated that 50% of EpCAM^+^ Hep3B single cells formed monoclones after 7 days' culture. The number of monoclones reached 80% after the second 7 days' culture. After the third 7 days' culture, all EpCAM^+^ Hep3B single cells formed monoclones. These results suggested that EpCAM^+^ cells could serve as a part of cancer stem/progenitor cells. WM130 exhibited a stronger inhibitory effect on the proliferation of EpCAM^+^ subpopulations than EpCAM^−^ subpopulations (Figure 4D). WM130 also concentration-dependently reduced the number of EpCAM^+^ cells in sorted EpCAM^+^ subpopulations as determined by flow cytometric analysis (Figure 4E). Consistently, WM130 inhibited the sphere formation of sorted EpCAM^+^ subpopulations. More specifically, WM130 treatment clearly reduced the number of primary spheres (Figure 4F). Furthermore, when WM130-treated primary spheres were cultured for the second passage in the absence of drug, the number of spherical colonies also significantly decreased compared with the control (Figure 4F). Moreover, WM130 also concentration-dependently reduced the size of single-cell derived EpCAM^+^ monoclones (Supplementary Figure S3D).

### Effect of WM130 on HCC xenograft growth

**Figure 5:**
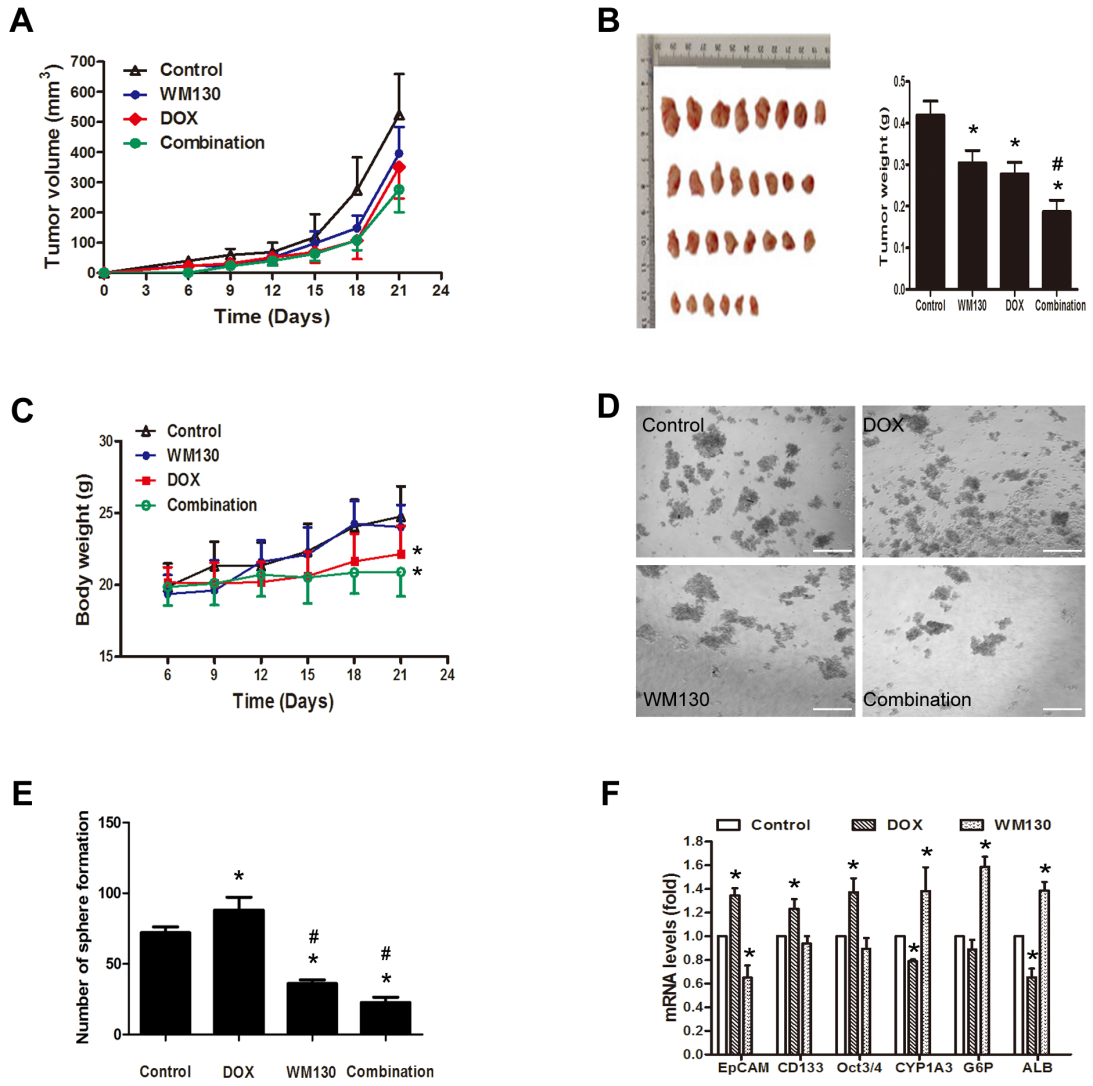
Effect of WM130 and DOX on tumor growth *in vivo* **A.** MHCC-LM3 tumor growth curves. **B.** Images and tumor weights of excised tumors. Similar results were observed in two separate experiments. A representative result was shown. N=6, *p<0.05 versus control and ^#^*p*<0.05 versus groups treated with either agent alone. **C.** Body weights of mice. N=6, *p<0.05 versus control or WM130 groups. **D** and **E.** Spheroid colony formation of cancer cells isolated from dissociated MHCC-LM3 tumors. N=6, *p<0.05 versus control and ^#^*p*<0.05 versus groups treated with either agent alone. Images of tumorsphere cultures are shown. Scale bar=100 μM. **F.** mRNA levels of stemness and liver-specific genes in tumor tissues. N=6, *p<0.05 versus control.

Using nude mice, we evaluated the anti-tumor effect of WM130 *in vivo*. WM130 markedly inhibited the growth of MHCC-LM3 xenografts. The combination of DOX and WM130 exerted a stronger inhibitory effect on tumor growth than either WM130 or DOX alone (Figure 5A). These results were in accordance with the weights of MHCC-LM3 xenografts at sacrifice (Figure 5B). The average body weight of WM130-treated mice did not vary significantly throughout the experiment, and WM130 did not aggravate DOX-induced weight loss (Figure 5C).

**Figure 6:**
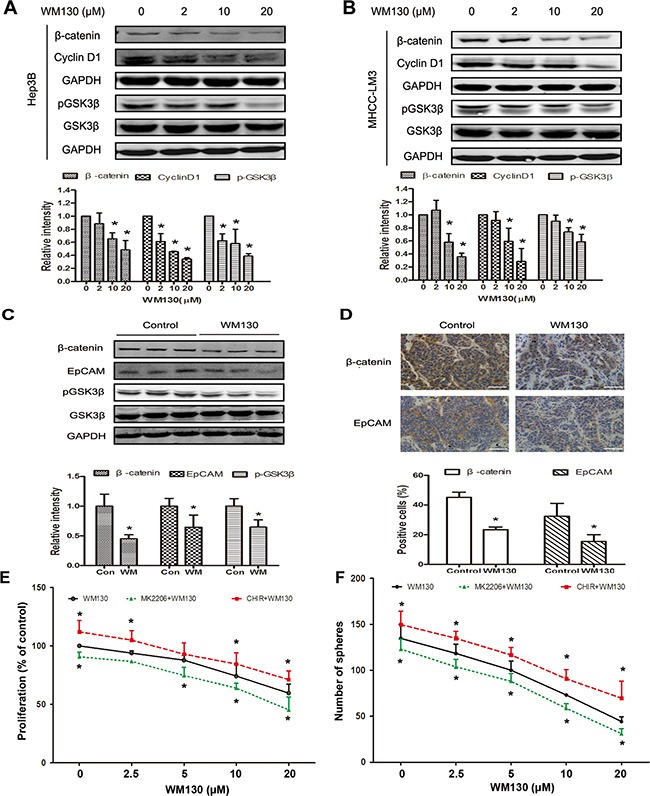
WM130 suppresses the GSK3β/β-catenin pathway *in vitro* and *in vivo* **A** and **B.** Western blot analysis of Hep3B and MHCC-LM3 cells treated with WM130 for 4 days. The band intensities were quantified. GAPDH was used as a loading control. N=3, *p<0.05 versus control. **C.** Western blot analysis of tumors from control- and WM130-treated mice. The representative results of three animals are shown. N=3, *p<0.05 versus control. **D.** Representative immunohistochemical staining of tumors from control- and WM130-treated mice. Scale bar=100 μM. β-catenin- and EpCAM-positive areas in tumor sections were quantified. N=3, *p< 0.05 versus control. **E.** Proliferation of EpCAM^+^ Hep3B cells treated with WM130, WM130 and MK2206 (0.5 μM), or WM130 and CHIR99021(2.5 μM). N=3, *p< 0.05 versus WM130. **F.** Sphere formation of EpCAM^+^ Hep3B cells treated withWM130, WM130 and MK2206 (0.5 μM), or WM130 and CHIR99021(2.5 μM). N=3, *p < 0.05 versus WM130.

We further investigated whether WM130 directly targeted hepatic cancer stem-like cells *in vivo.* The number of sphere-forming cells decreased in WM130-treated tumors compared with the control tumors. In contrast, the number increased in DOX-treated tumors. Of note, WM130 further reduced the number of tumor sphere-forming cells when administered in combination with DOX (Figure 5D and 5E). Further investigation revealed that WM130 administration remarkably decreased the levels of *EpCAM* mRNA and protein in tumor xenografts (Figure 5F, Figure 6C and D), which was accompanied by the increased expression of *CYP1A3*, *G-6-P* and *ALB*. In contrast, DOX increased the expression of *EpCAM, CD133* and *Oct3/4*, but decreased the expression of *CYP1A3* and *ALB* (Figure 5F).

In addition, the colony formation ability of MHCC-LM3 cells from WM130-treated mice and WM130 plus DOX-treated mice significantly decreased compared with the control (Supplementary Figure S4A). Of note, MHCC-LM3 cells from DOX-treated mice displayed higher proliferation ability than those from control mice, and the cells were resistant to DOX but sensitive to WM130 upon *in vitro* treatment (Supplementary Figure S4B).

### WM130 suppresses the GSK3β/β-catenin pathway in hepatoma cells *in vitro* and *in vivo*

Further mechanism study revealed that WM130 markedly decreased the phosphorylation of GSK3β (Ser9) in Hep3B and MHCC-LM3 cells. Moreover, WM130 reduced the protein level of β-catenin in parallel with decreased expression of *Cyclin D1*, a Wnt/β-catenin target gene (Figure 6A and 6B). Consistent results were obtained *in vivo* in the MHCC-LM3 tumor xenografts. More specifically, WM130 administration notably reduced the phosphorylation of GSK3β (Ser9) and decreased the expression of β-catenin and its target EpCAM in the xenografts, as evidenced by western blotting and immunostaining results (Figure 6C and 6D).

We have previously shown that WM130 suppressed the AKT pathway in HCC cells [36]. To investigate if the inhibition of the AKT/GSK3β pathway contributed to the inhibitory actions of WM130 on hepatic CSCs, EpCAM^+^ cells were treated with series concentrations of WM130 and specific AKT inhibitor MK2206 (0.5 μM) or GSK3β inhibitor CHIR99021 (2.5 μM). As shown in Figure 6E and 6F, treatment with MK2206 resulted in slight inhibition of cell proliferation and sphere formation, and co-treatment of cells with WM130 and MK2206 result in a greater inhibition than that observed with WM130 alone. In contrast, CHIR99021 increased cell proliferation and sphere formation, and antagonized WM130's effects on inhibiting the growth and sphere formation of EpCAM^+^ cells.

## DISCUSSION

CSCs are believed to be responsible for HCC growth and relapse, and the cure of HCC requires the eradication of hepatic cancer stem cells [14, 17]. WM130 has been previously shown to inhibit hepatoma Huh-7 cell xenograft growth. This effect is associated with the inhibition of cell proliferation, invasion and migration, and the induction of apoptosis in HCC cells [36]. Nevertheless, the effect of WM130 on CSCs remains unknown. In the current study, by using hepatoma sphere cells and the sorted hepatoma EpCAM^+^ cells, we evaluated the inhibitory effect of WM130 on HCC CSCs.

Through sphere culture technique, we found that three types of Hep3B, MHCC-LM3 and MHCC-97H spheres exhibited CSC features, including self-renewal, clonogenicity, expression of CSC biomarkers EpCAM and CD133, and resistance to chemotherapeutic drug such as DOX. The CSC features of Hep3B spheres were confirmed by the findings in *in vivo* tumorigenicity assay. Using these sphere cells as models, we found that WM130 concentration-dependently reduced the subpopulation expressing CSC biomarkers EpCAM/CD133. Moreover, the WM130-treated sphere cells exhibited weaker tumorigenicity in mice compared with the control. CSCs being resistant to chemotherapy, our results also showed that WM130 could inhibit DOX-resistant cells and eliminate the remarkable increase of EpCAM^+^ cells in the resistant cells. Of note, the WM130-induced reduction of cancer stem-like cells (EpCAM/CD133 positive) appeared to be associated with the suppression of self-renewal capability and the promotion of differentiation from CSCs to hepatocytes. More specifically, WM130 induced a marked decrease not only for primary spheres, but also for spheres in the subsequent two passages without WM130 treatment, indicating the suppression of self-renewal capability. Additionally, the expression of stem cell biomarkers was reduced and the expression of mature hepatocyte biomarkers was increased in the WM130-treated HCC cells, indicating the promotion of differentiation from CSCs to hepatocytes. Interestingly, in contrast to DOX, WM130 preferred to inhibit sphere cell proliferation, colony formation and *EpCAM* mRNA expression. Consistently, WM130 had a stronger inhibitory effect on EpCAM^+^ subpopulations than EpCAM^−^ subpopulations. Collectively, these data suggest that WM130 can selectively inhibit cancer stem-like cells (CSLCs).

In a xenograft model, we substantiated our observations *in vitro* that WM130 preferred to inhibit CSLCs. WM130 alone had an inhibitory effect on tumor growth and markedly reduced the number of surviving CSLCs. In contrast, DOX inhibited tumor growth but increased the number of surviving CSLCs. Notably, cotreatment with DOX and WM130 resulted in a higher inhibition of tumor growth than treatment alone. Moreover, the number of surviving CSLCs in DOX and WM130-cotreated mice was even lower, which was consistent with our *in vitro* finding that WM130 suppressed DOX-resistant HCC cells, especially EpCAM^+^ cells. It would be more definitive to assess the ability of residual cancer stem cells to initiate tumors upon secondary implantation in NOD/SCID mice [21, 37]. Notwithstanding this limitation, this study does suggest that WM130 preferentially targeted hepatic CSLCs rather than the bulk cell population.

Wnt/β-catenin signaling is one of the key pathways for CSC self-renewal [38]. The expression of the hepatic stem cell biomarker EpCAM is regulated by Wnt/β-catenin signaling, and β-catenin degradation or β-catenin inhibitors could reduce *EpCAM* gene expression [14, 39]. We have previously shown that WM130 could inhibit the growth of tumor xenografts and suppress EGFR and PTEN/AKT signaling pathways [36]. In the current study, we showed that WM130 could reduce the protein levels of β-catenin and its target gene *Cyclin D1* in hepatoma cells. Importantly, downregulation of β-catenin and EpCAM proteins was clearly observed in tumor xenografts of WM130-treated groups. These results suggested that WM130 could downregulate the Wnt/β-catenin self-renewal pathway. The level of intracellular β-catenin is regulated by AKT substrate GSK3β, which phosphorylates β-catenin and promotes ubiquitin-proteasome degradation of the phosphorylated β-catenin [40]. Phosphorylation of GSK3β at Ser9 results in its inhibition, thereby the stabilization of β-catenin [41, 42]. Our data clearly demonstrated that WM130 decreased the phosphorylation of GSK3β (Ser9) in HCC cells and tumor xenografts, suggesting that WM130-induced β-catenin degradation was possibly mediated by the activation of GSK3β. Recently, an AKT inhibitor has been demonstrated to potently reduce the number of brain cancer stem cells. This effect was associated with a preferential induction of apoptosis and a suppression of neurosphere formation [43]. To conclude that the inhibition of the AKT/GSK3β pathway by WM130 contributes to its inhibitory effect on hepatic CSCs, we used specific AKT inhibitor MK2206 and GSK3β inhibitor CHIR99021. The results verified our speculation. MK2206 inhibited, whereas CHIR99021 increased EpCAM^+^ cell proliferation and sphere formation. Moreover, co-treatment of cells with WM130 and MK2206 had an additive effect, while CHIR99021 antagonised WM130's effect. Taken together, our data suggest that the inhibitory effect of WM130 on hepatic CSLCs was mediated, at least partly, by inhibiting the AKT/GSK3β/β-catenin signaling pathway.

Collectively, this study demonstrates that WM130 preferentially inhibits hepatic CSLCs and reduces HCC xenograft growth. These beneficial effects were associated with the downregulation of the AKT/GSK3β/β-catenin signaling pathway. We also show that the combination of WM130 and DOX exhibits a stronger anti-cancer efficacy than either treatment alone. These findings provide a strong rationale for the development of WM130 as a novel drug candidate for HCC therapy.

## MATERIALS AND METHODS

### Cell lines and reagents

Human HCC cell lines Hep3B, MHCC-LM3, and MHCC-97H were kindly provided by the Department of Gastroenterology, Shanghai Changzheng Hospital, Second Military Medical University. All cells were grown in Dulbecco's modified Eagle's medium (DMEM) supplemented with 10% fetal bovine serum (Gibco BRL, NY), 100 U/ml penicillin, and 100 U/ml streptomycin in a 37°C incubator containing 5% CO_2_.

WM130 (>98% purity) was synthesized in our laboratory, dissolved at a concentration of 40 mmol/L in 1 mmol/L acetate acid as a stock solution, and diluted with medium or saline before use. Doxorubicin (DOX) hydrochloride was obtained from Main Luck Pharmaceuticals Inc. (Shenzhen, China). Recombinant human basic fibroblast growth factor (FGF), recombinant human epidermal growth factor (EGF), and DMEM/F-12 were purchased from PeproTech (Rocky Hill, NJ). B27 (50×) and Insulin-Transferrin-Selenium (ITS, 100×) were from Gibco BRL. L-glutamine (100×) was from Invitrogen (Carlsbad, CA). Anti-CD133 (AC133)-PE and anti-CD326 (EpCAM)-APC antibodies, and isotype-matched mouse anti-IgG1-PE and anti-IgG1-APC were from Miltenyi Biotec (North Rhine-Westphalia, Germany). Antibodies against β-catenin, GSK-3β, phospho-GSK-3β (Ser9), and EpCAM were purchased from Cell Signaling Technology (Beverly, MA). Antibodies against Cyclin D1 and glyceraldehyde phosphate dehydrogenase (GAPDH) were from Santa Cruz Biotechnology (Santa Cruz, CA). IRDye680-conjugated anti-rabbit and anti-mouse secondary antibodies were purchased from Rockland (Gilbertsville, PA). AKT inhibitor MK2206 and GSK3β inhibitor CHIR99021 were purchased from Selleck Chemicals (Shanghai, China).

### Cell proliferation and colony formation assays

HCC cells were cultured at a density of 3 ×10^3^ cells per well in 96-well plates (NEST Biotech, Wuxi, China) overnight and then exposed to WM130. After 72 h of culture, cell proliferation was assessed using a Cell Counting Kit-8 (CCK8, Dojindo Laboratories, Tokyo, Japan), and the 50% inhibition concentration (IC_50_) was calculated. For the colony formation assay, cells were seeded at a density of 1×10^3^ cells per well in 6-well plates (NEST Biotech) and treated with or without WM130. Culture medium was changed every 3-5 days. 17-21 days later, the colonies were stained with Coomassie brilliant blue and photographed. The total number of colonies (>50 cells/colony) was counted.

### Spheroid formation assay

Spheroid culture was performed as described previously [11, 21] with a slight modification. Briefly, HCC spheres were cultured in DMEM/F-12 supplemented with FGF (20 ng/mL), EGF (20 ng/mL), B27 (1×), ITS (1×), and L-glutamine (1×). For WM130 treatment, primary sphere cells were dissociated with trypsin to a single cell suspension and seeded in 96-well ultra-low attachment plates (Corning, Lowell, MA) at a density of 1×10^3^ cells/well. The cells were then exposed to various concentrations of WM130 for 7 days. The second and third passages of cells were grown for 7 days in the absence of WM130. The number of spheres (>30 μm in diameter) was counted, and spheres were photographed under a phase contrast microscope (Olympus, Tokyo, Japan).

### Isolation of EpCAM^+^ and EpCAM^−^ HCC cells

To obtain EpCAM^+^ and EpCAM^−^ enriched cell populations, cells were firstly incubated with EpCAM antibodies conjugated with magnetic beads with biotin binded. Separation was carried out by using a magnetic column (Miltenyi Biotec). EpCAM^+^ cells were maintained in their undifferentiated state using stem cell medium. EpCAM^−^ cells were maintained in their differentiated state with DMEM medium. To examine the effect of WM130, EpCAM^−^ medium was changed to EpCAM^+^ medium before treatment with WM130.

### Flow cytometric analysis

Cells were harvested and incubated with anti-AC133-PE and anti-EpCAM-APC antibodies. Isotype-matched mouse anti-IgG1-PE and anti-IgG1-APC were used for controls. Samples were analyzed with a FACSCalibur flow cytometer (BD Biosciences, San Jose, CA).

### Quantitative real-time RT-PCR

Total RNA was extracted using an RNAsimple Total RNA Kit (Tiangen, Shanghai, China). cDNA was synthesized from 1 μg total RNA using a Prime Script RT reagent Kit (TaKaRa, Dalian, China). Real-time PCR was performed using a SYBR Green PCR Kit (TaKaRa, Dalian, China) with the primers listed in Supplementary Table S1. The mRNA expression was normalized against β-actin mRNA levels.

### Western blotting

Proteins were extracted, and approximately 20 μg of total proteins were subjected to SDS-polyacrylamide gel electrophoresis, and transferred to a nitrocellulose membrane. The membrane was incubated with primary antibodies against β-catenin (1:1000), Cyclin D1 (1:1000), GSK-3β (1:500), phospho-GSK-3β (1:1000), or GAPDH (1:3000), followed by IRDye680-conjugated anti-rabbit or anti-mouse secondary antibodies (1:5000). Detection was performed with an Odyssey infrared imaging system (LI-COR Biotechnology, Nebraska).

### Tumor xenograft model

Four-week-old male Balb/c nude mice were purchased from Shanghai SLAC Laboratory Animal Co. Ltd (Shanghai, China). Hep3B spheres were treated with or without 10 μmol/L WM130 for 4 days and then allowed to recover and expand for another 4 days without treatment prior to injection. The WM130-treated cells (1×10^5^) were mixed with Matrigel (BD Biosciences) and subcutaneously injected into the flank of each mouse. To avoid individual differences, an equal number of vehicle-treated control cells were subcutaneously injected into the opposite flank of the same mouse. Tumor growth was monitored for 45 days after cell injection.

For *in vivo* drug treatments, 1×10^6^ MHCC-LM3 cells were subcutaneously injected into nude mice. Drug treatments were initiated at 24 h after cell injection. Animals were administered either saline, WM130 (20 mg/kg, orally by gavage daily), DOX (6 mg/kg, intraperitoneal injection, once a week), or WM130 in combination with DOX for 3 weeks. The tumor size was measured with a caliper as the longest surface length (mm; L) and width (mm; W). Tumor volume (mm^3^; V) was calculated as V=1/2×LW^2^. All mice were sacrificed, and tumor tissues were excised and weighted at the end of experiments.

A portion of the tumor tissues was dissociated enzymatically to obtain a single cell suspension for spheroid formation assay, proliferation assay and colony formation assay, and the remaining tumor tissues were used for real-time RT-PCR analysis, western blotting and immunostaining. All procedures involving animals and their care were approved by our institutional Animal Care Committee in accordance with the institutional guidelines for animal experiments.

### Immunohistochemical staining

Tumor tissue sections were fixed by immersion in 10% paraformaldehyde diluted in phosphate buffered saline, dehydrated in graded ethylic alcohol solutions, and embedded in paraffin. Immunohistochemical examinations were carried out to detect the expression of β-catenin and EpCAM (1:500) with a primary mouse monoclonal antibody and horseradish-peroxidase-conjugated goat anti-mouse IgG (Gene Tech, Shanghai, China) as the secondary antibody. All staining was visualized and photographed under an ordinary optical microscope. Areas of immunohistochemically positive and negative cells were determined using a computer-assisted automated image analyzer (Qwin Leica) as described previously [34].

### Statistical analysis

Results are expressed as the mean ± standard deviation. Statistical analyses were performed using one-way analysis of variance or the two-tailed Student's t test. *p* < 0.05 was considered as the minimum level of significance.

## SUPPLEMENTARY FIGURES AND TABLE



## Abbreviations

CSCs: cancer stem cells
HCC: hepatocellular carcinoma
CSLCs: cancer stem-like cells
EpCAM: epithelial cell adhesion molecule
DOX: doxorubicin
GSK3β: glycogen synthase kinase 3β
AKT: protein kinase B (PKB)
