# Supplementary material for: WM130 preferentially inhibits hepatic cancer stem-like cells by suppressing AKT/GSK3β/β-catenin signaling pathway
Source: Oncotarget. 2016 Oct 22;7(48):79544–56. doi: 10.18632/oncotarget.12822 (PMC5346734; doi:10.18632/oncotarget.12822)
Supplement: Supplementary file 1 [file oncotarget-07-79544-s001.pdf]

# WM130 preferentially inhibits hepatic cancer stem-like cells by suppressing AKT/GSK3β/β-catenin signaling pathway

## SUPPLEMENTARY FIGURES AND TABLE

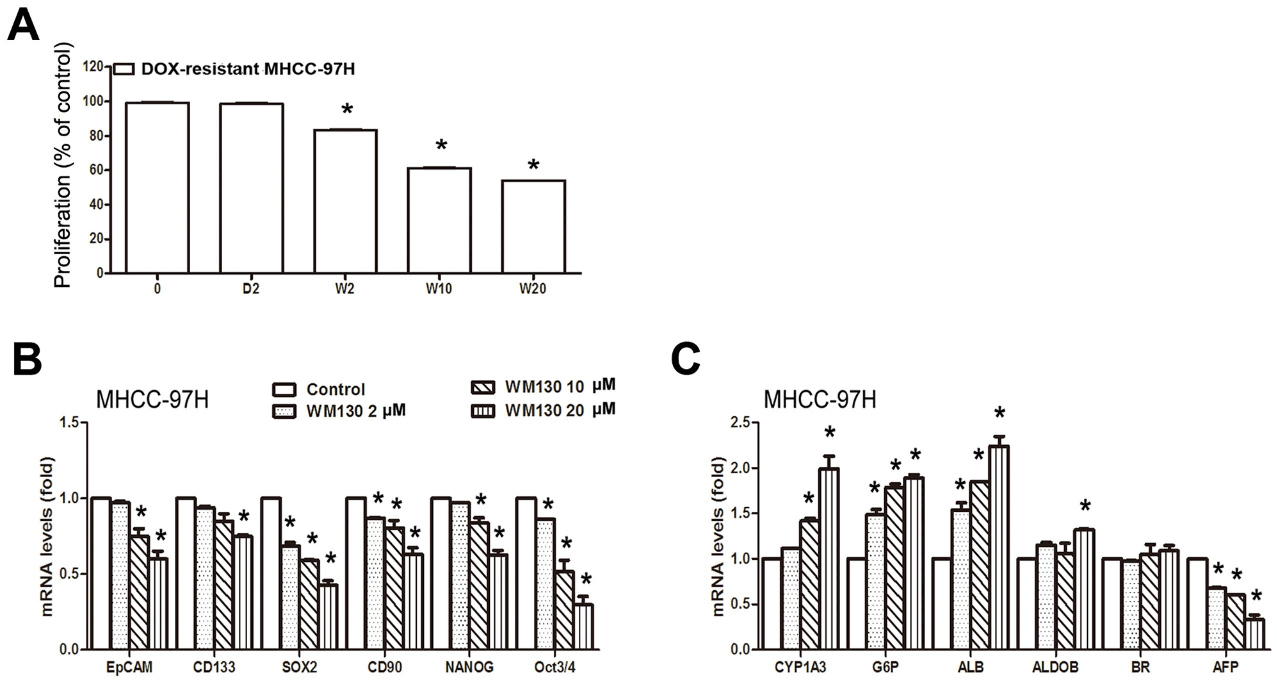


**Supplementary Figure S1: The effect of WM130 on DOX-resistant MHCC-97H cells and the expression of genes relevant to stemness and differentiation of hepatic stem/progenitor cells. A.** DOX-resistant MHCC-97H cells remained sensitive to WM130. N=3, $^*p<0.05$ versus control. **B** and **C.** MHCC-97H cells were treated with WM130 for 24 h. The expression of "stemness" and liver-specific genes was detected by real-time RT-PCR. The mRNA levels were normalized against β-actin and are relative to the control. N=3, $^*p<0.05$ versus control.

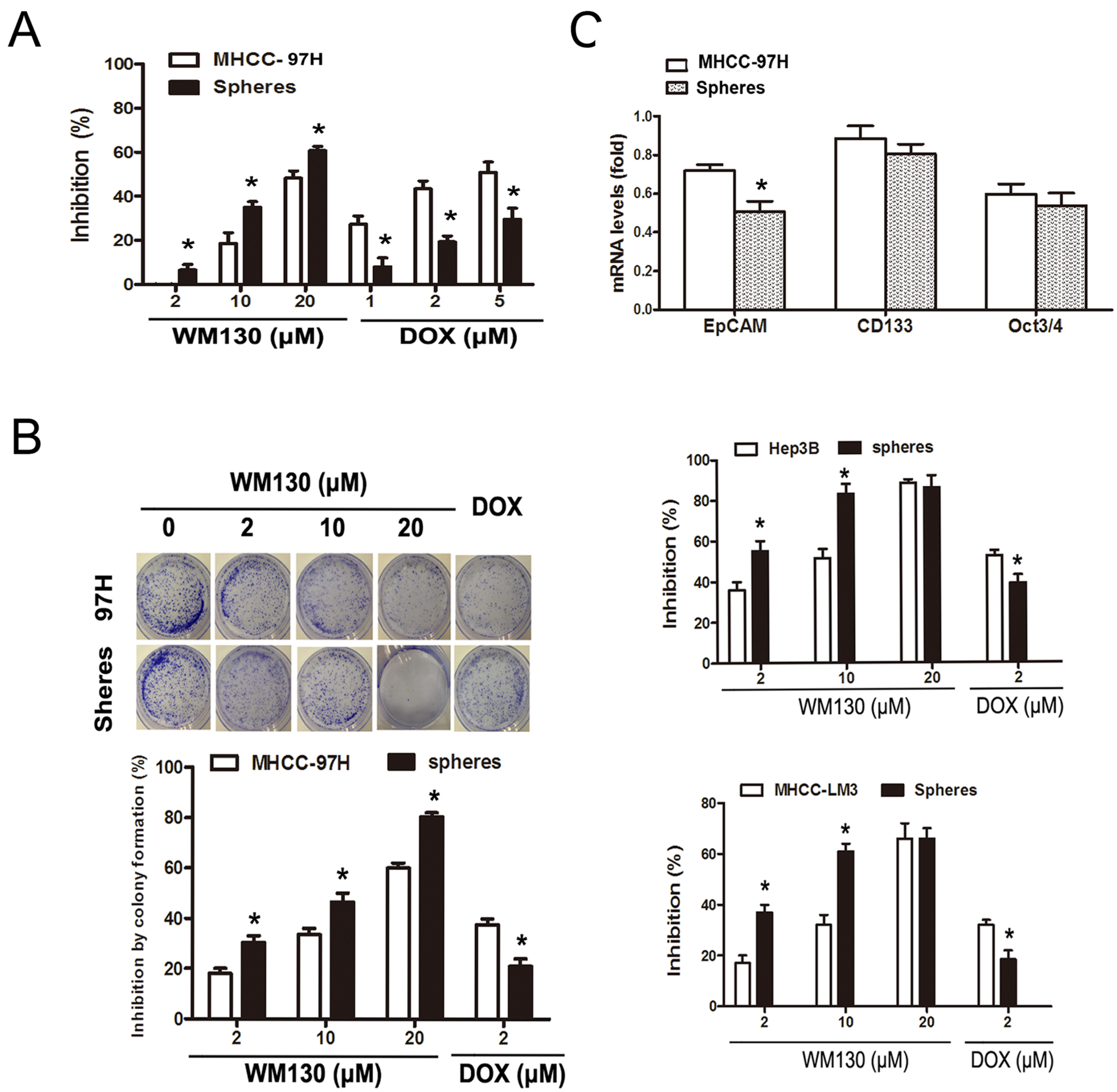


**Supplementary Figure S2: Preferential inhibition of MHCC-97H sphere cells by WM130. A.** Growth inhibitory effects of WM130 and DOX on MHCC-97H sphere cells compared with their corresponding parental cells. N=3, **p*<0.05 versus control. **B.** Inhibition of colony formation by WM130 and DOX. N=3, **p*<0.05 versus control. **C.** WM130 preferentially decreased EpCAM expression in spheroids. N=3, **p*<0.05 versus control.

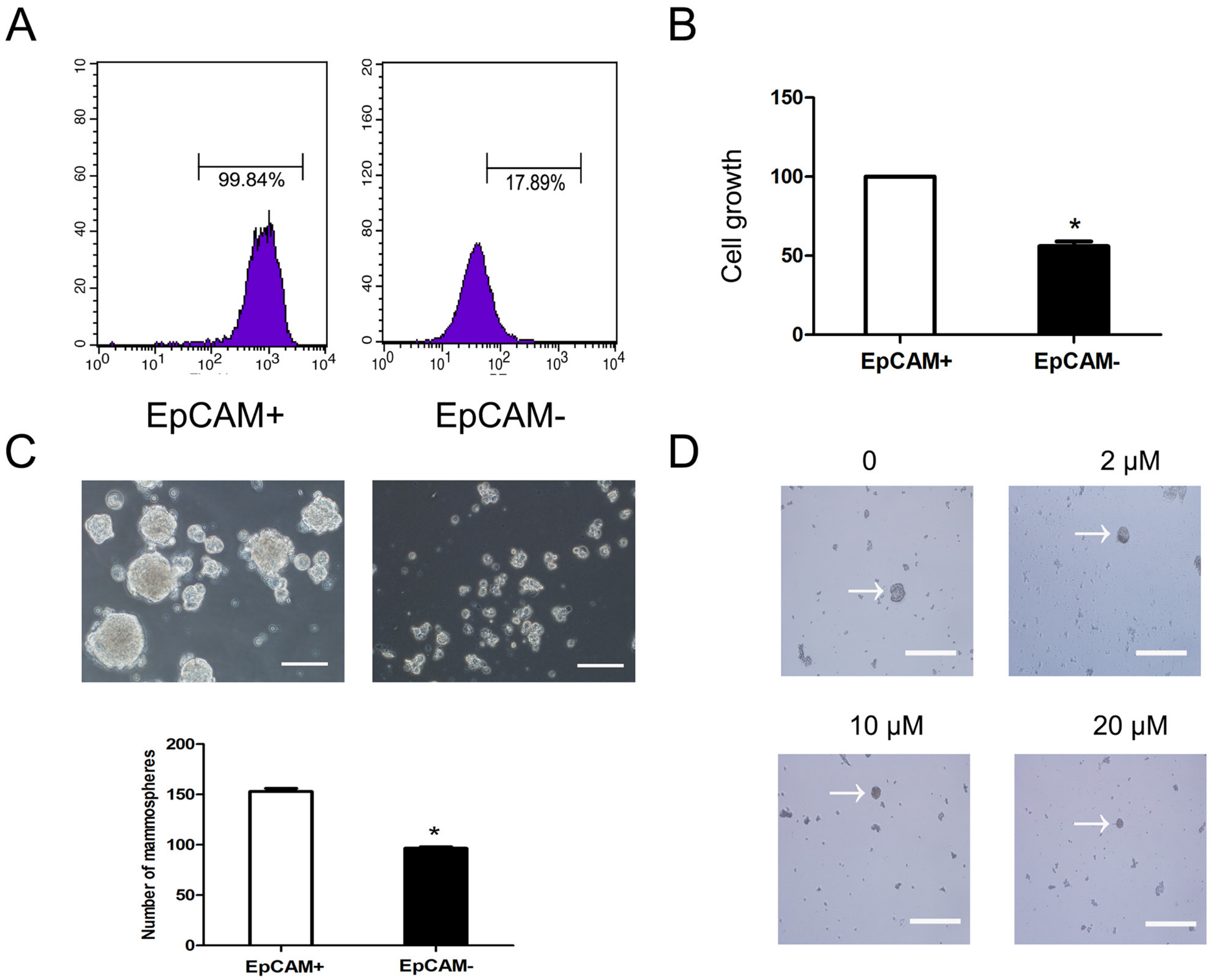


**Supplementary Figure S3: The sorted EpCAM+ Hep3B cells exhibits features of cancer stem/progenitor cells. A.** Purity of the sorted of $EpCAM^+$ and $EpCAM^-$ Hep3B cells. **B.** The sorted $EpCAM^+$ Hep3B cells exhibited stronger proliferation ability than $EpCAM^-$ cells. N=3, $*p<0.05$ versus $EpCAM^+$ Hep3B cells. **C.** The sorted $EpCAM^+$ Hep3B cells exhibited better sphere forming ability than $EpCAM^-$ cells. N=3, $*p<0.05$ versus $EpCAM^+$ Hep3B cells. Scale bar=100 μM. **D.** WM130 noticeably reduced the size of single-cell derived $EpCAM^+$ monoclones in a concentration dependant manner. Scale bar=100 μM.

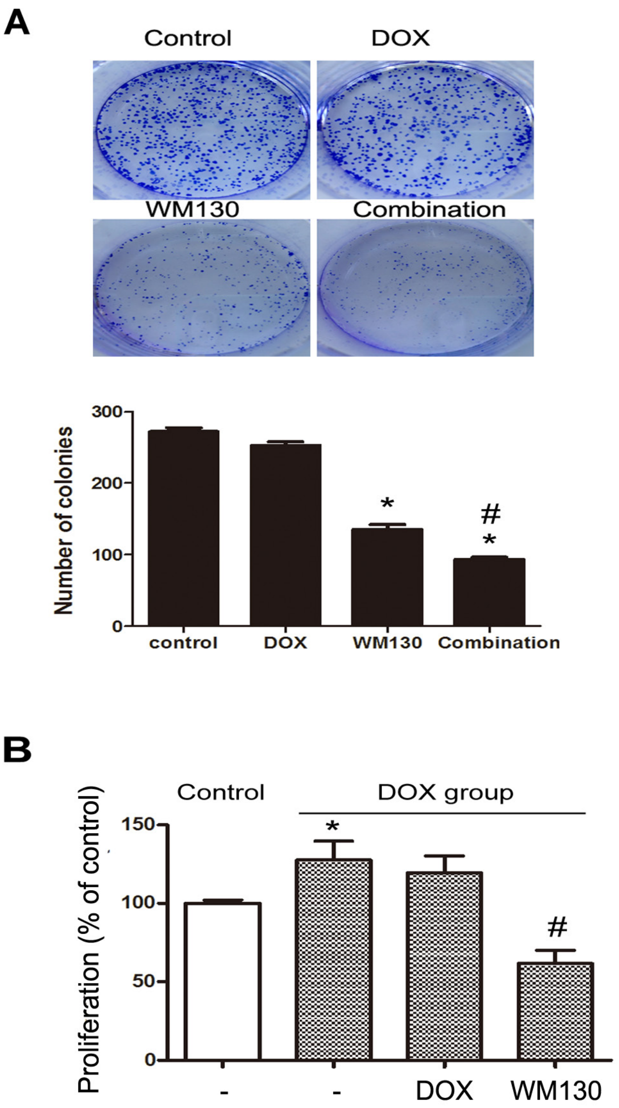


**Supplementary Figure S4: Colony formation and proliferation of cancer cells isolated from dissociated MHCC-LM3 tumors. A.** Colony formation of cancer cells isolated from dissociated MHCC-LM3 tumors. N=6, **p*<0.05 versus control and #P<0.05 versus groups treated with either agent alone. **B.** Cancer cells from DOX-treated mice showed higher proliferation compared with cells from control-treated mice and were *in vitro* resistant to DOX (2 μmol/L) but sensitive to WM130 (10 μmol/L) as determined by CCK8 assays. N=3, **p*<0.05 versus control-treated mice and # *p*<0.05 versus untreated control.

**Supplementary Table S1: Sequence of primers**

| Gene | Sequence |
|---|---|
| *β-actin* | F: 5′-ACCCACACTGTGCCCATCTATG-3′<br>R: 5′-AGAGTACTTGCGCTCAGGAGGA-3′ |
| *EpCAM* | F: 5’-GCTCTGAGCGAGTGAGAACCT-3’<br>R: 5’-GACCAGGATCCAGATCCAGTTG-5’ |
| *CD133* | F: 5′-ACATGAAAAGACCTGGGGG-3′<br>R: 5′-GATCTGGTGTCCCAGCATG-3′ |
| *CD90* | F: 5′-CGGAAGACCCCAGTCCA-3′<br>R: 5′-ACGAAGGCTCTGGTCCACTA-3′ |
| *G-6-P* | F: 5′-GGCTCCATGACTGTGGGATC-3′<br>R: 5′-TTCAGCTGCACAGCCCAGAA-3′ |
| *ALB* | F: 5′-AGCCTAAGGCAGCTTGACTT-3′<br>R: 5′-CTCGATGAACTTCGGGATGA -3′ |
| *BR* | F: 5′-ACAAGGTGCTGCGGGAATCA-3’<br>R: 5′-ACTGGTGGGAGGGGTAGGTG-3’ |
| *CYP1A3* | F: 5′-CTGGCCTCTGCCATCTTCTG-3′<br>R: 5′-TTAGCCTCCTTGCTCACATGC-3′ |
| *Oct3/4* | F: 5′-CGACCATCTGCCGCTTTGAG-3′<br>R: 5′-CCCCCTGTCCCCCATTCCTA-3′ |
| *Nanog* | F: 5’-CCCTGATTCTCCCACCAGTCC-3’<br>R: 3’-AGTCGGGTTCACCAGGCATCC-3’ |
| *Sox2* | F: 5′-GCGAACCATCTCTGTGGTCT-3′<br>R: 5′-GGAAAGTTGGGATCGAACAA-3′ |
| *AFP* | F: 5’-TACGTCCCTCCACCATTTC-3’<br>R: 5’-ATCCTGGTCTTTGCAGCACT-3’ |
